# Supplementary figures and images for: How does Sec63 affect the conformation of Sec61 in yeast?
Source: PLoS Comput Biol. 2021 Mar 29;17(3):e1008855. doi: 10.1371/journal.pcbi.1008855 (PMC8031780; doi:10.1371/journal.pcbi.1008855)

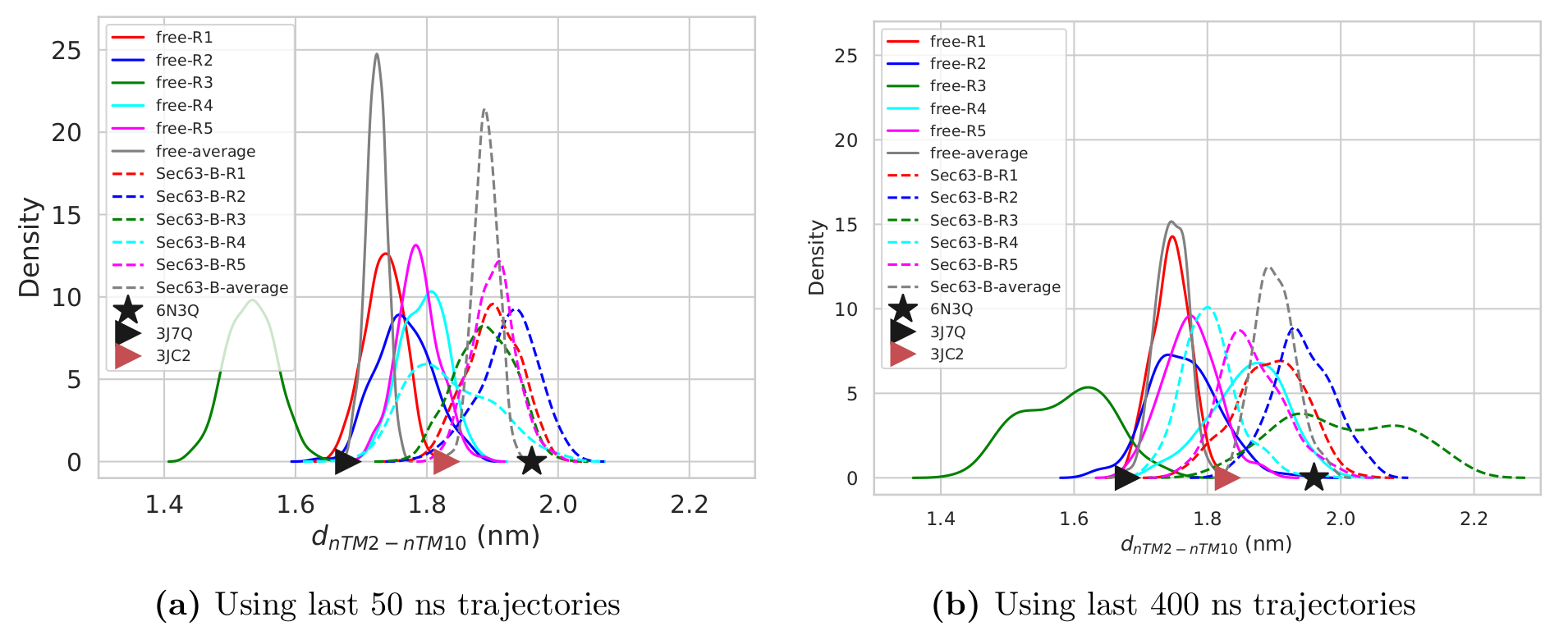

Supplement: S1 Fig — Solid and dashed lines represent ‘free’ (free; without Sec63) and ‘Sec63-B’ (Sec63-bound) states, respectively. Black star, black triangular and red triangular symbols represent the values in the experimental cryo-EM structures of Sec complex, idle-state ribosome-Sec61 complex, and open-state ribosome-Sec61 complex, respectively. (TIF) [file pcbi.1008855.s001.tif]

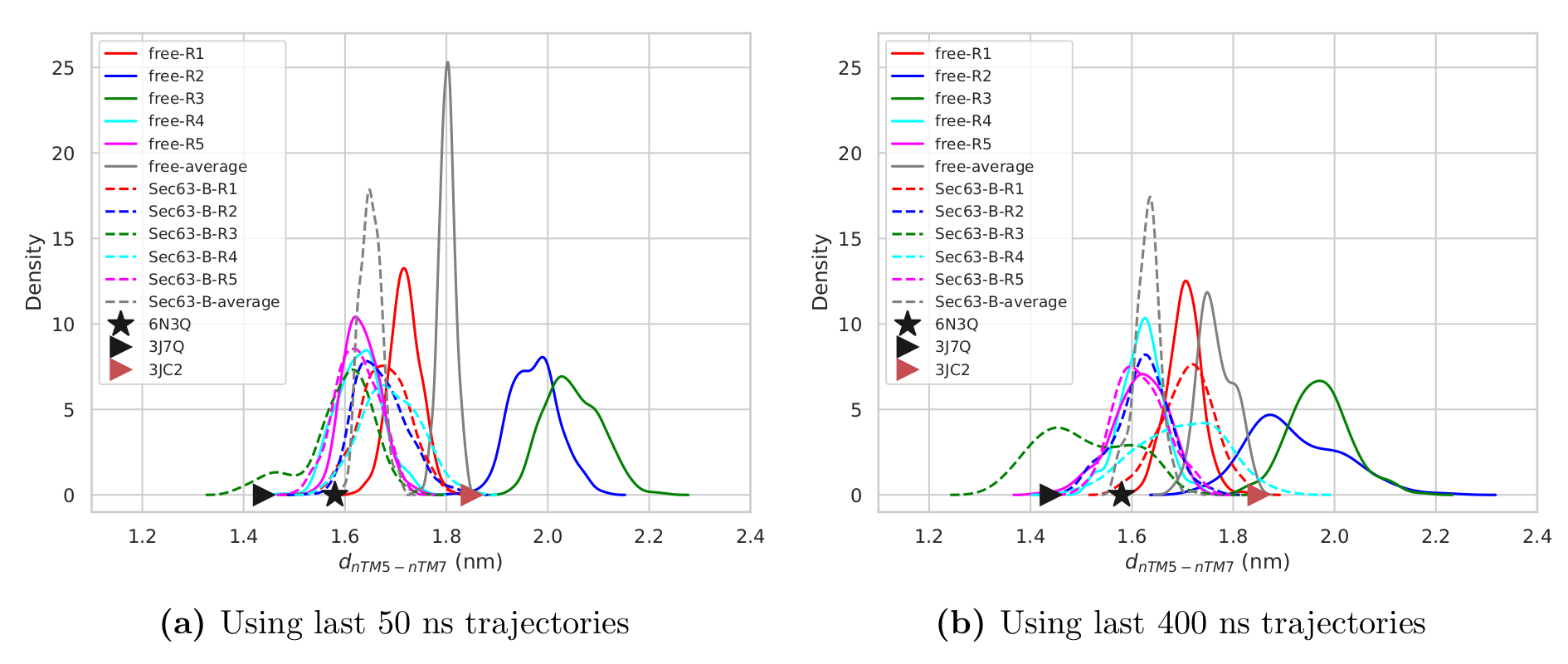

Supplement: S2 Fig — Solid and dashed lines represent ‘free’ (free; without Sec63) and ‘Sec63-B’ (Sec63-bound) states, respectively. Black star, black triangular and red triangular symbols represent the values in the experimental cryo-EM structures of Sec complex, idle-state ribosome-Sec61 complex and open-state ribosome-Sec61 complex, respectively. (TIF) [file pcbi.1008855.s002.tif]

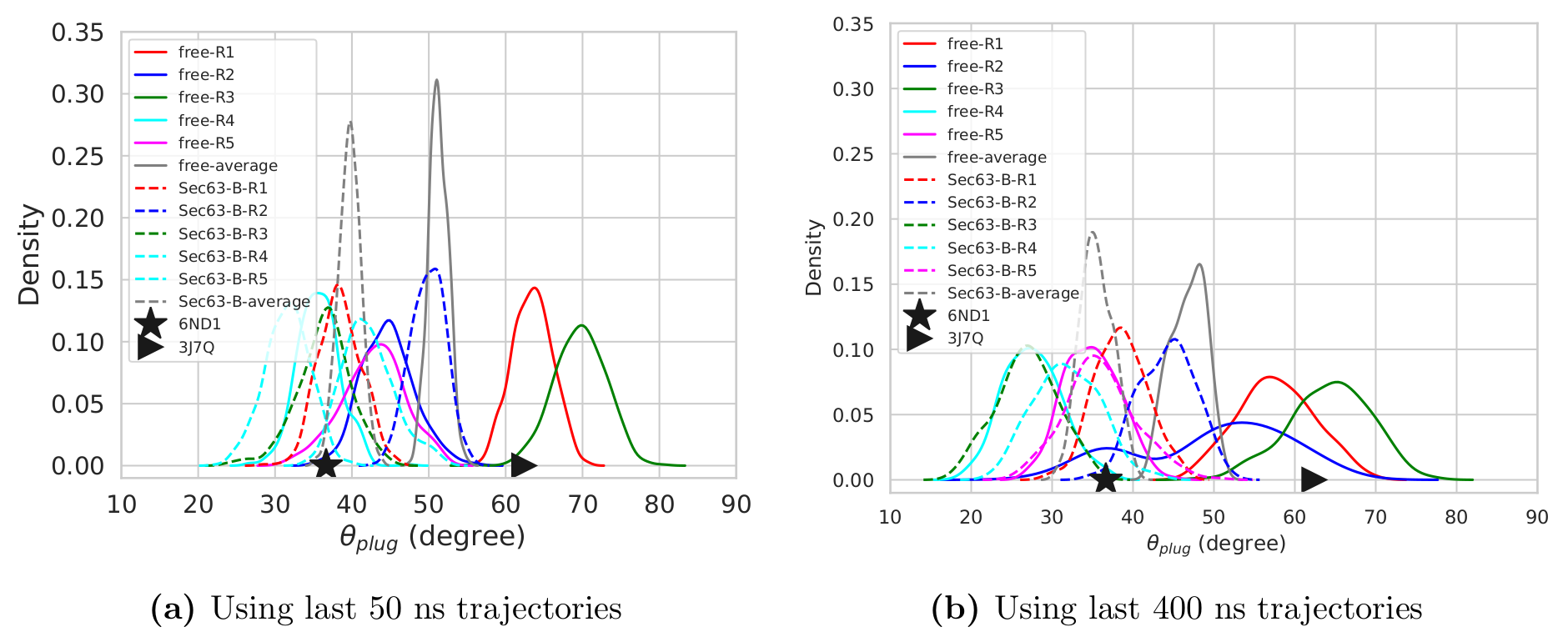

Supplement: S3 Fig — Solid and dashed lines represent simulations of the ‘free’ (free) and ‘Sec63-bound’ (Sec63-B) states, respectively. Black star and triangular symbols represent values in the experimental cryo-EM structures of Sec complex and ribosome-Sec61 complex, respectively. The plug region is missing in the cryo-EM structure of the open-state ribosome-Sec61 complex (PDB-ID:3JC2). (TIF) [file pcbi.1008855.s003.tif]

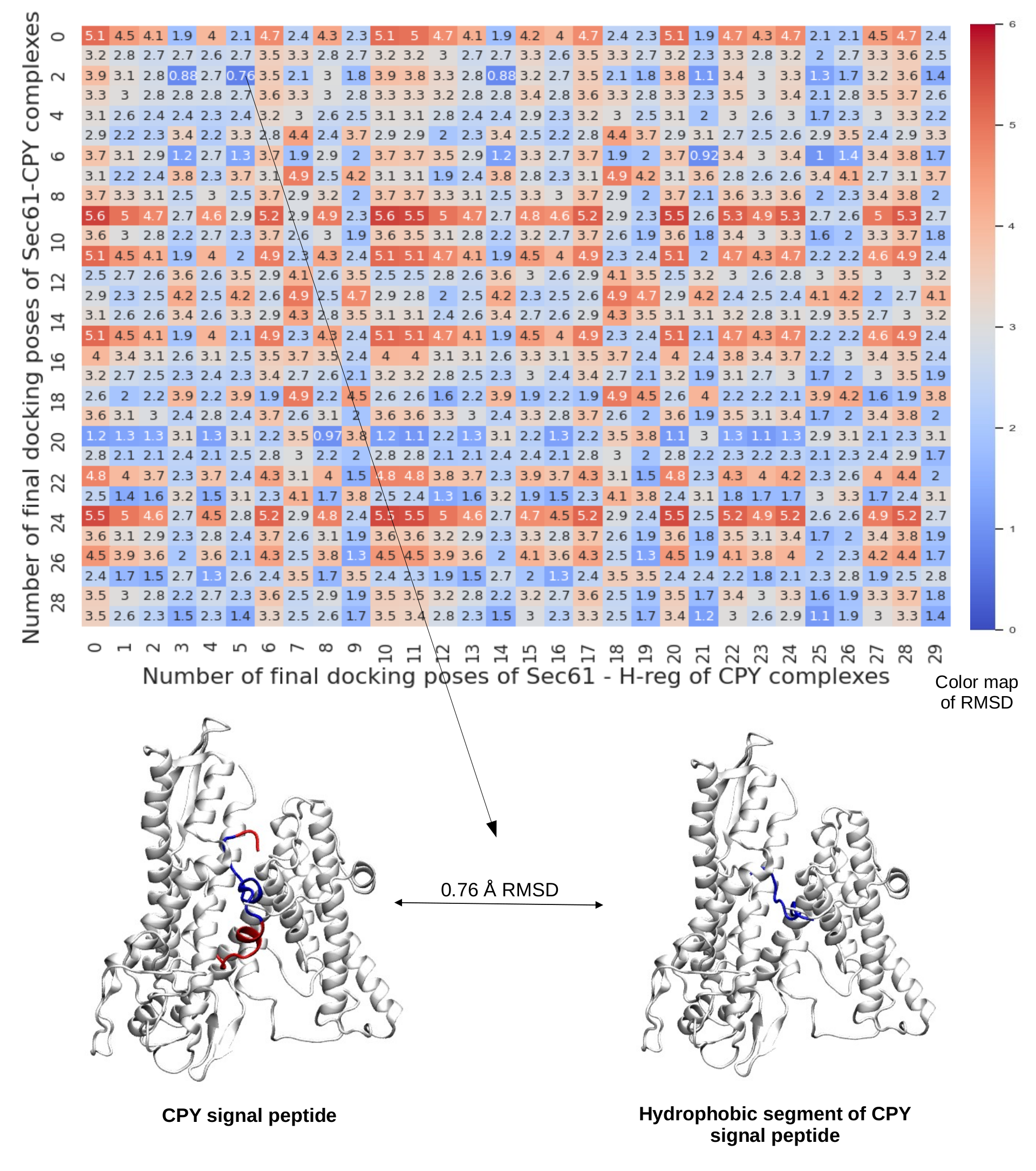

Supplement: S4 Fig — The bottom panel shows the conformation with lowest RMSD value. Sec61 (white), hydrophobic region of CPY (blue) and other regions of CPY (red) are shown in white, blue, and red, respectively. (TIF) [file pcbi.1008855.s004.tif]

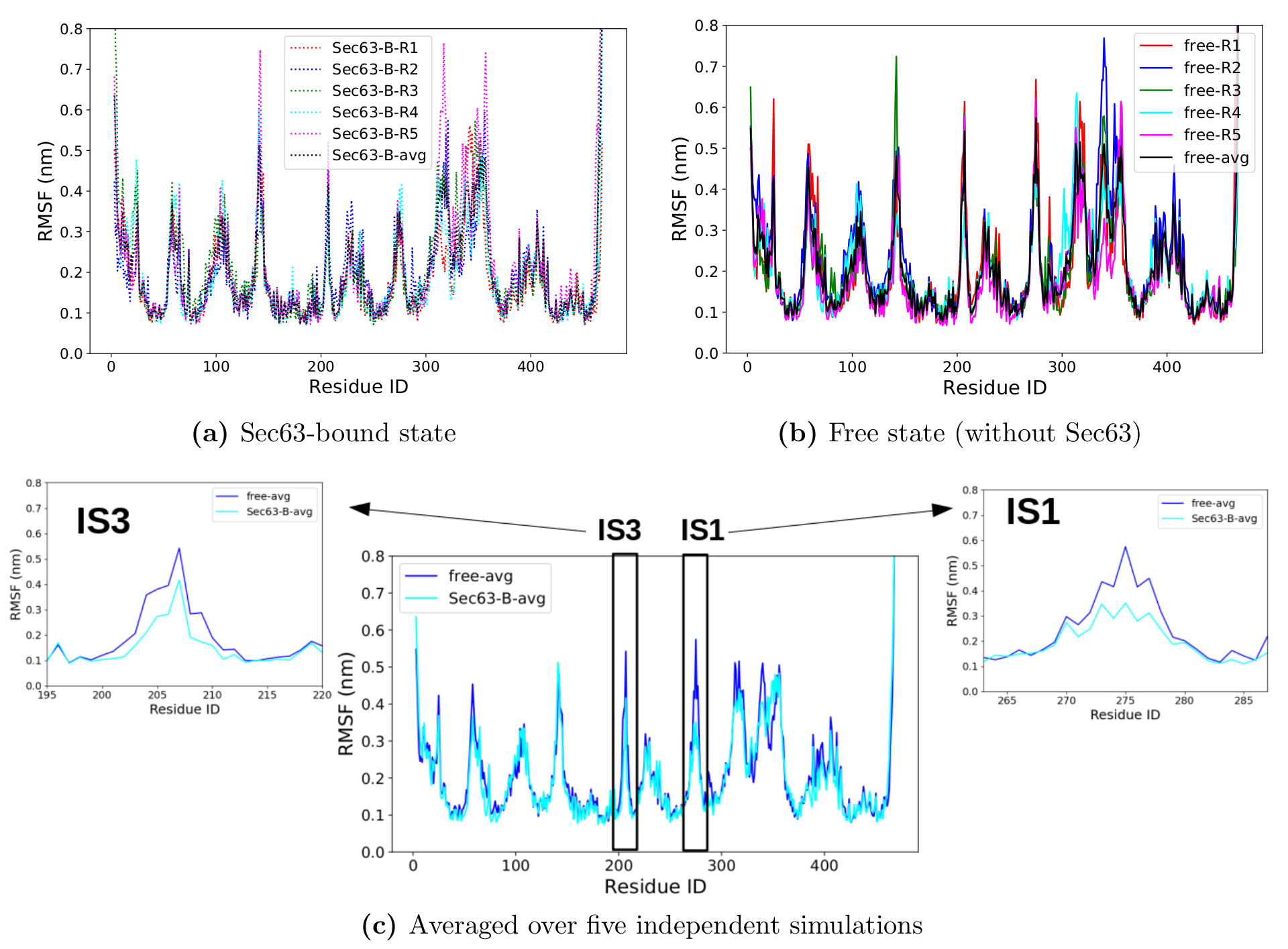

Supplement: S5 Fig — (A) and (B) represent RMSF values in the individual simulations at Sec63-bound and free state, respectively. (C) shows averages of the five simulations. IS3 and IS1 regions are magnified. IS3 (hypergeometric p-value = 0.023, threshold: RMSF of Gly206 > 0.55 nm) and IS1 (hypergeometric p-value = 0.004, threshold: RMSF of Arg275 > 0.5 nm) regions are significantly more flexible in free state simulations compared to the Sec63-bound state. (TIF) [file pcbi.1008855.s005.tif]

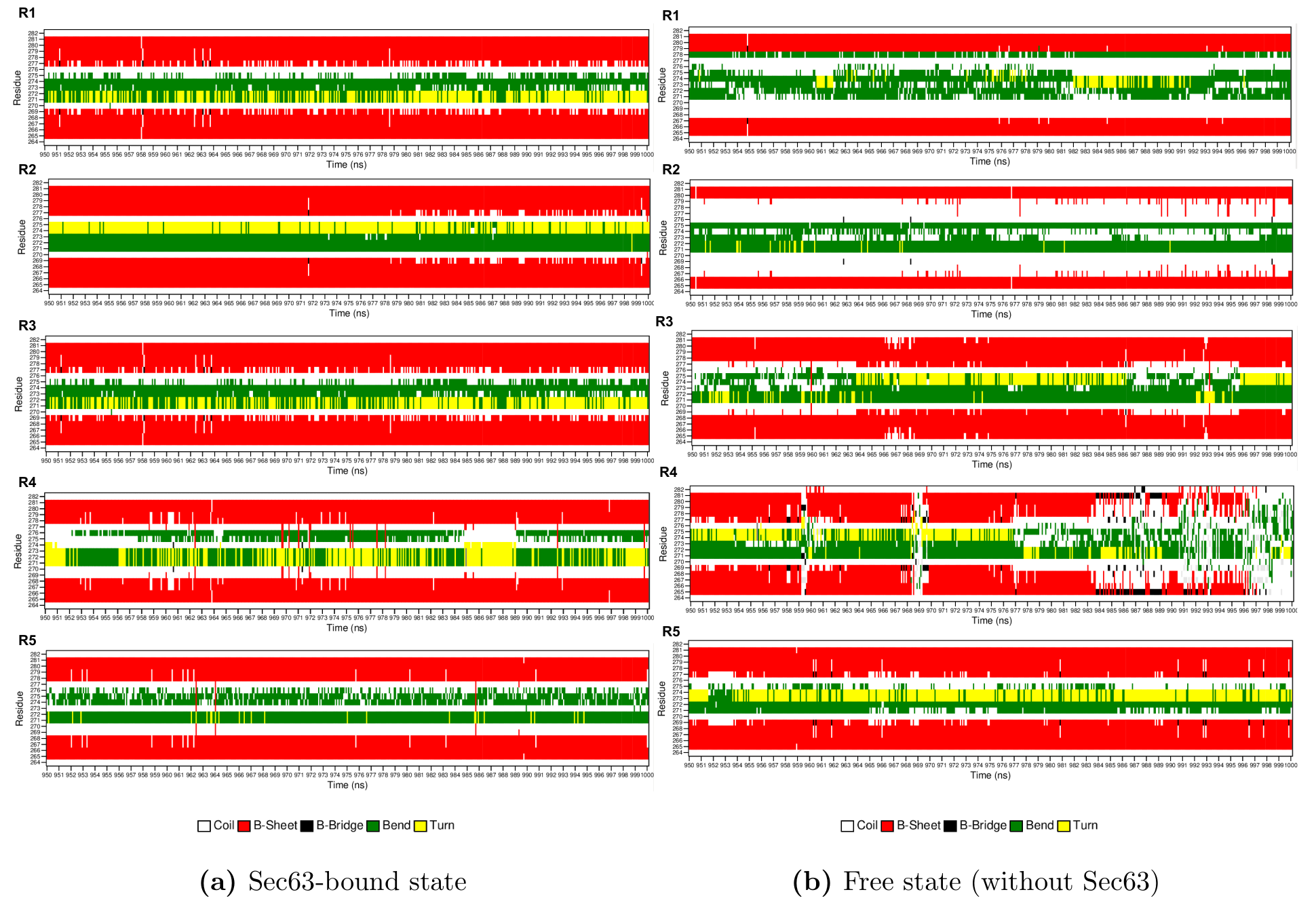

Supplement: S6 Fig — (A) Sec63-bound state (B) Free state (without Sec63). (TIF) [file pcbi.1008855.s006.tif]

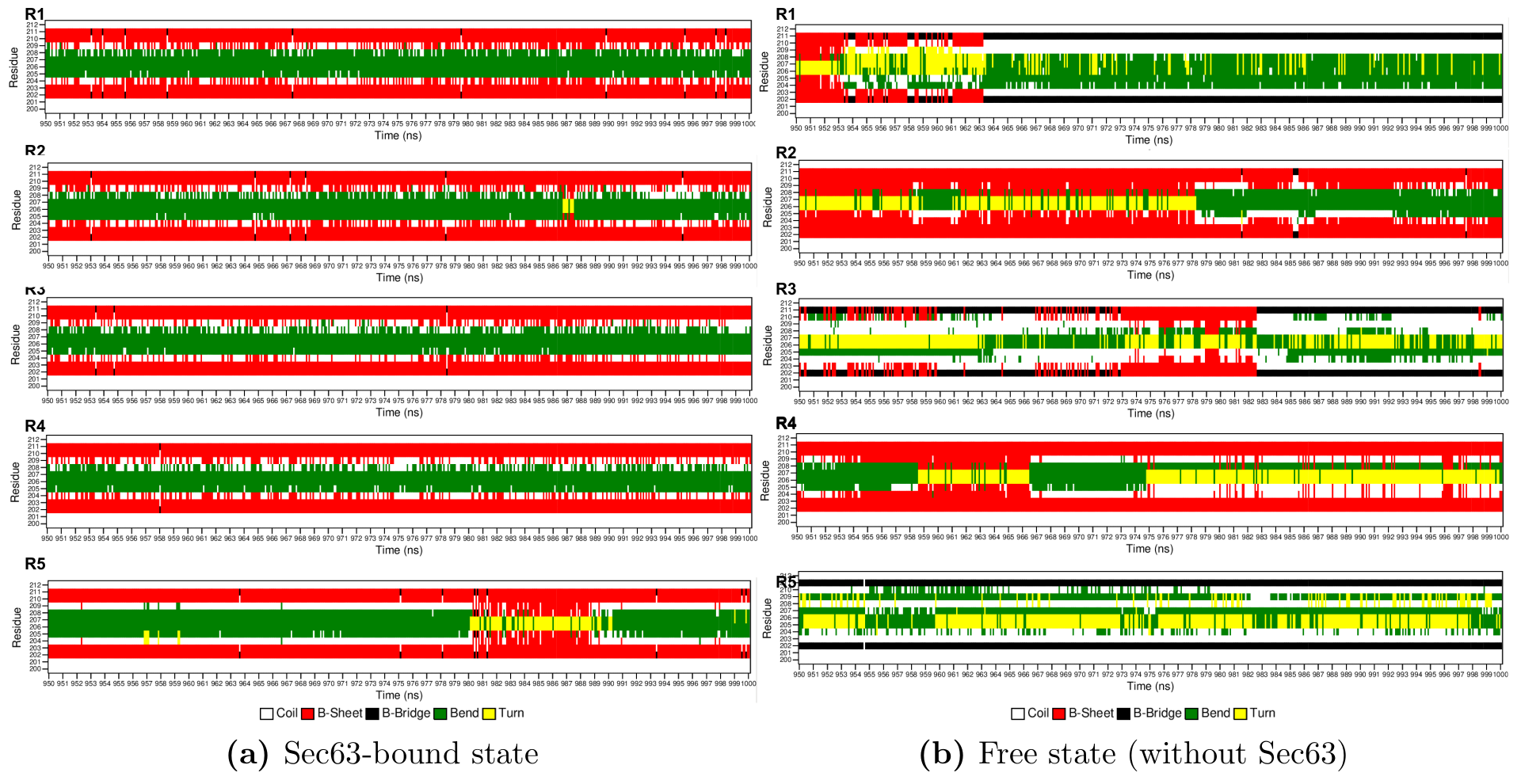

Supplement: S7 Fig — (A) Sec63-bound state (B) Free state (without Sec63). (TIF) [file pcbi.1008855.s007.tif]

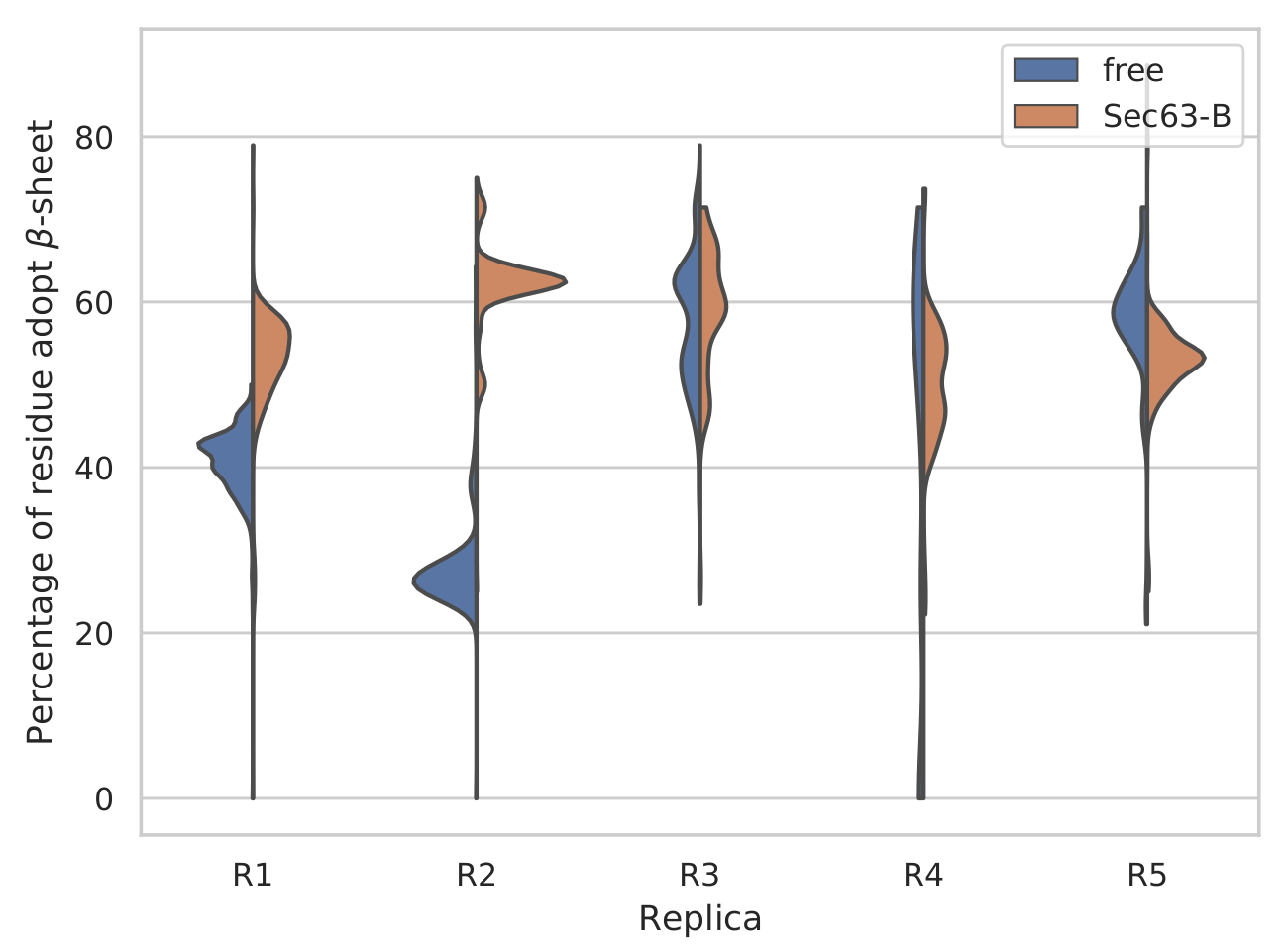

Supplement: S8 Fig — ‘Sec63-B’ and ‘free’ denote Sec63-bound and free states, respectively. The value is 65% in both cryo-EM structures of the Sec complex and of the ribosome associated Sec61 complex. (TIF) [file pcbi.1008855.s008.tif]

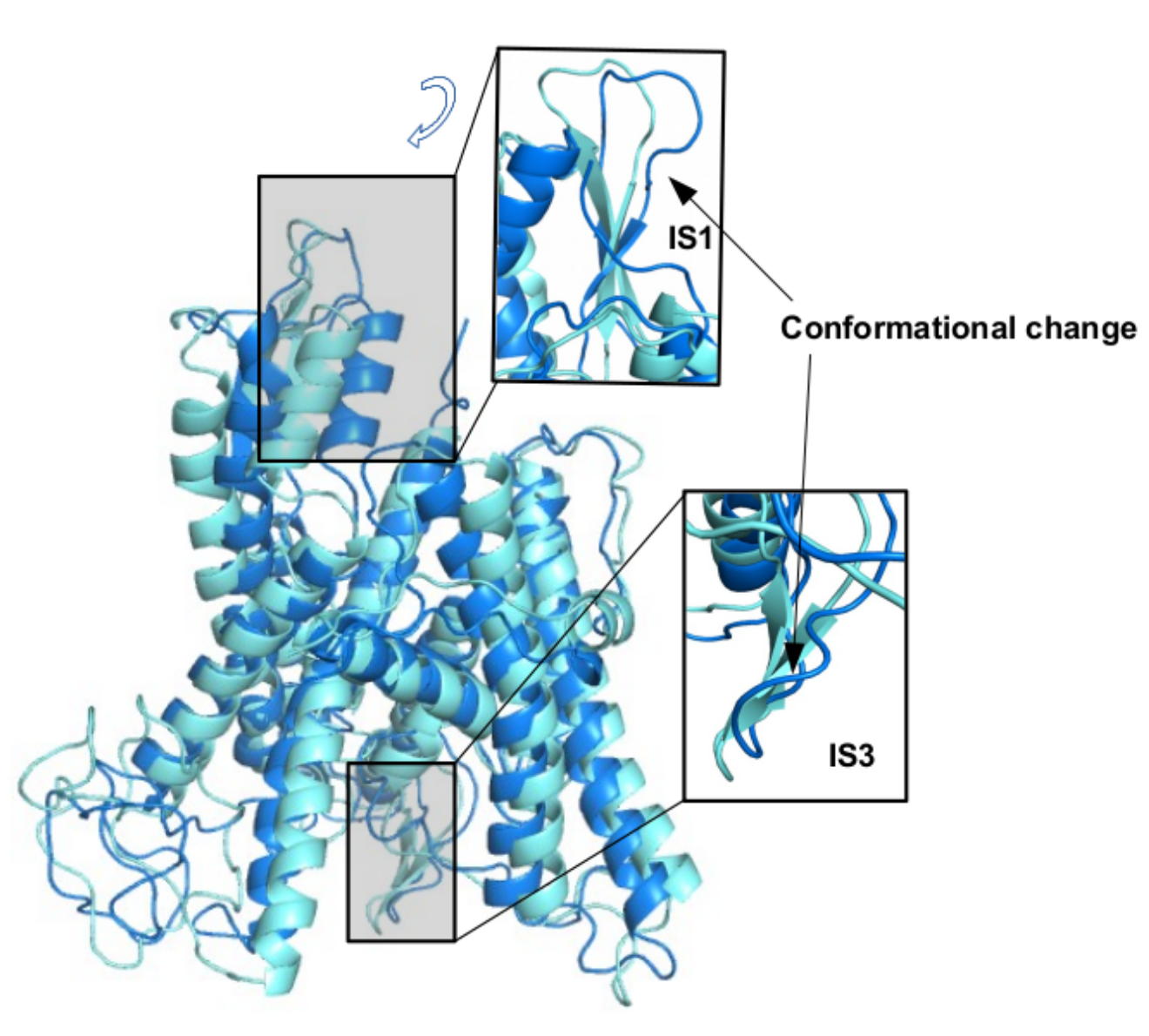

Supplement: S9 Fig — (TIF) [file pcbi.1008855.s009.tif]

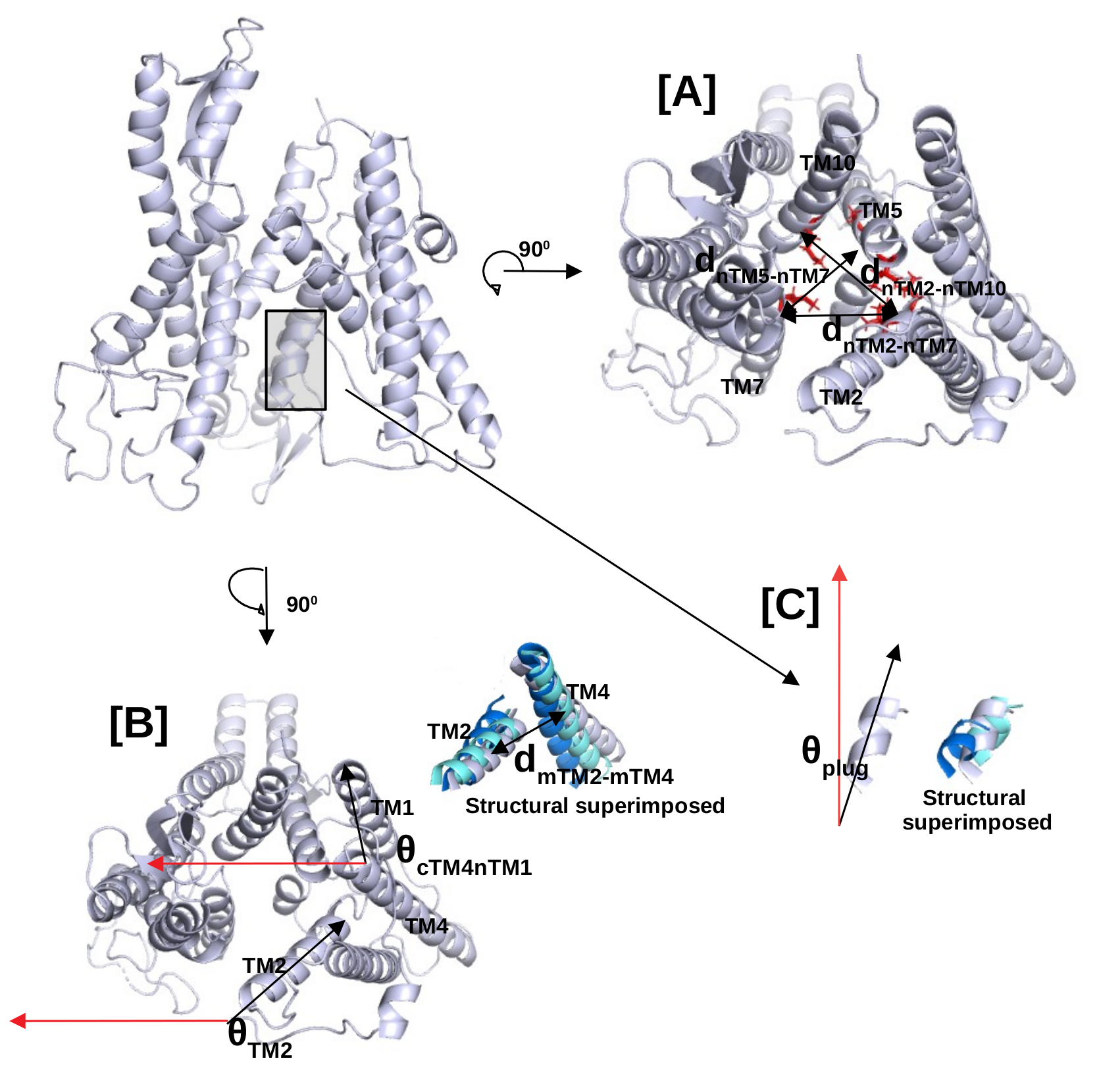

Supplement: S10 Fig — (A) dnTM2–nTM10, dnTM5_nTM7 and dnTM2_nTM7 are the distances between the center-of-mass of the N-terminal helical turns of TM2 (S83-I86) & TM10 (S447-M450), TM5 (I181-F184) & TM7 (P292-L295) and TM2 (S83-I86) & TM7 (P292-L295), respectively. (B) The orientation of TM2 is defined by the angle between the TM2 helical axis (black) and the vector parallel to the lipid layer oriented along the x-axis (red), (θTM2). The distance between TM2 and TM4 helices is measured by the distance between COM of Cα atoms of the middle helical turn of TM2 (S89-F92) and TM4 (M158-S161). The orientation of the C-terminus of TM4 (C168-L171) with respect to the N-terminus of TM1 (N30-L33) is described by the angle θcTM4nTM1. (C) The orientation of the plug helix with respect to the lipid bilayer is measured by the angle between the plug helical axis (black) and the vector normal to the lipid layer (red), θPlug. In superimposed structures blue, and cyan colors represent free and Sec63-bound states, respectively. (TIF) [file pcbi.1008855.s010.tif]

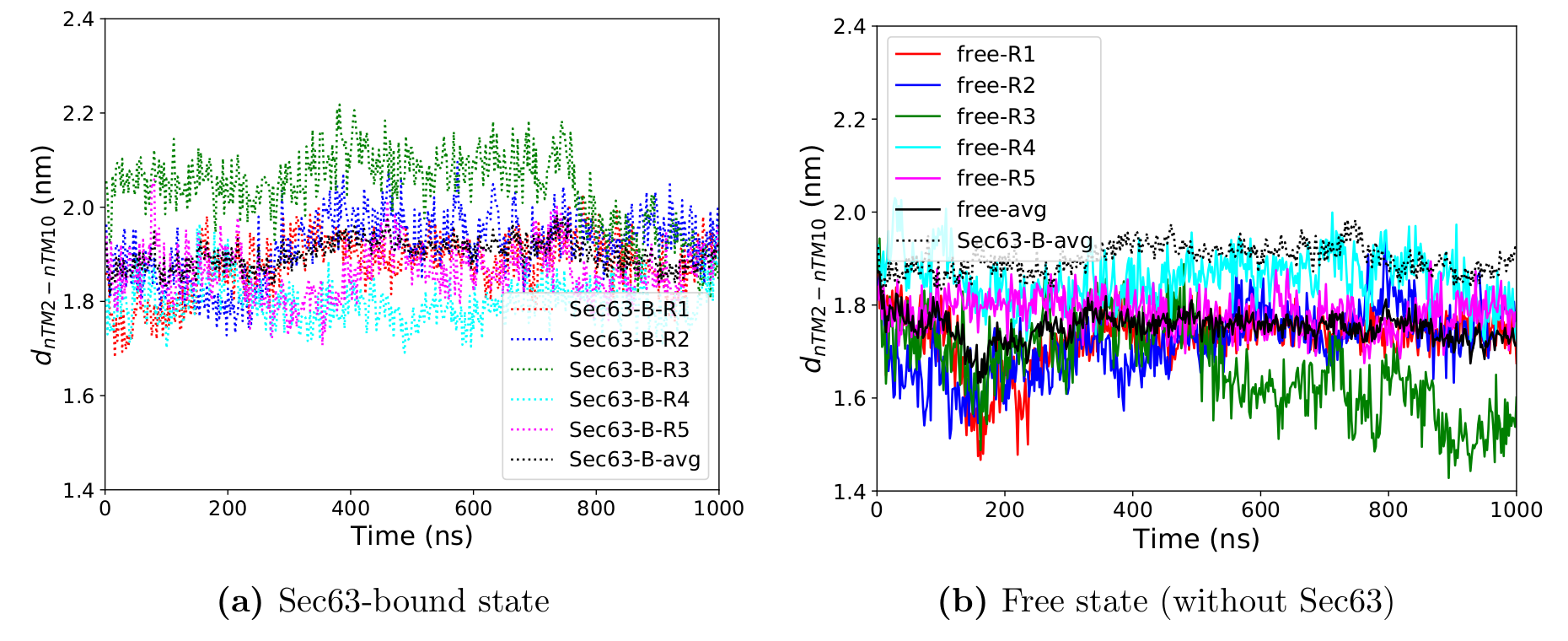

Supplement: S11 Fig — ‘Sec63-B’ and ‘free’ denote Sec63-bound and free states. The right panel also includes Sec63-B-avg from the left panel to illustrate the deviation between Sec63-B-avg and free-avg. (see also S12, S14, S16 and S18 Figs). (A) Sec63-bound state (B) Free state (without Sec63). (TIF) [file pcbi.1008855.s011.tif]

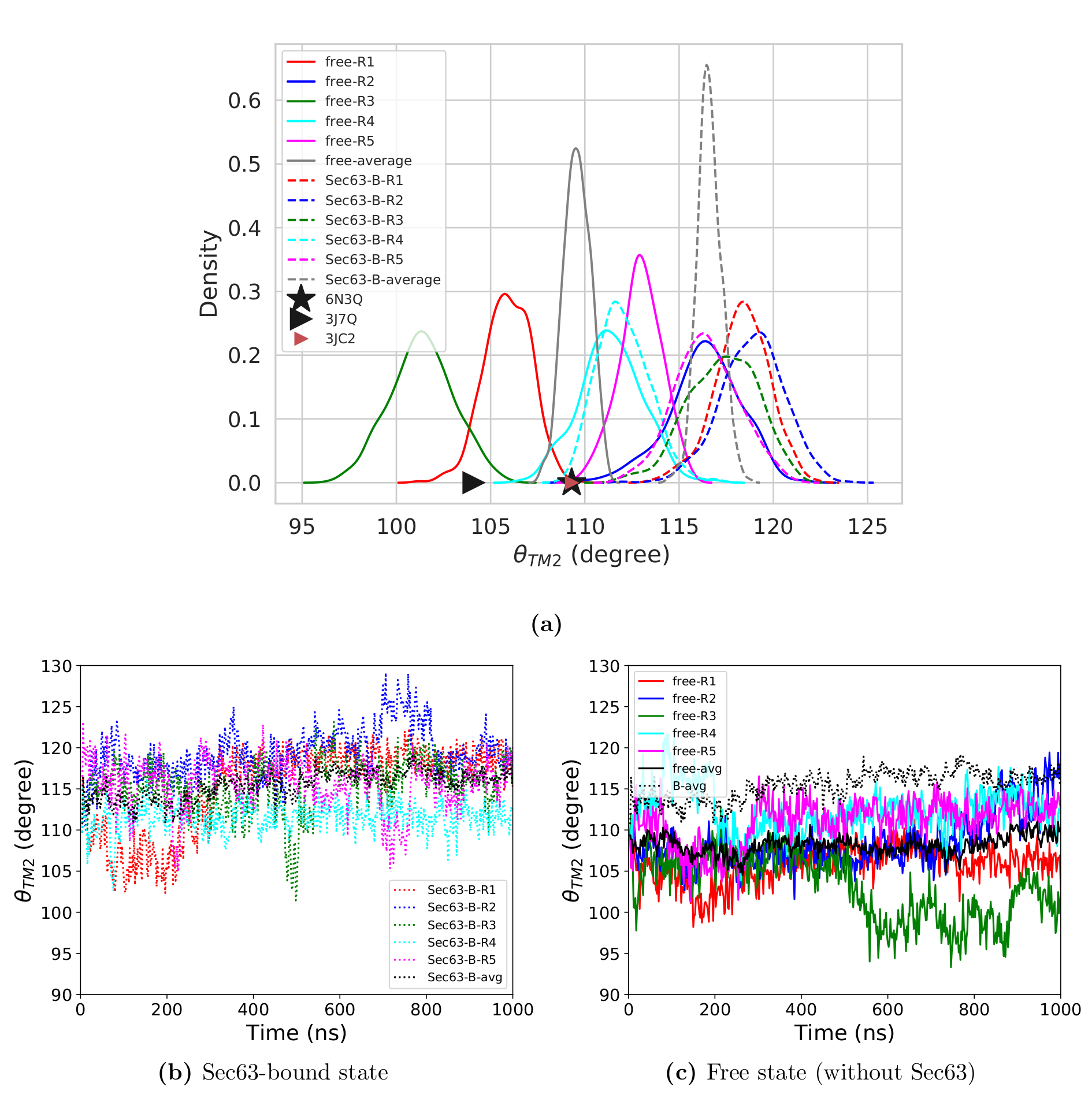

Supplement: S12 Fig — (A) Probability distributions of θTM2 values obtained from the last 50 ns of five MD simulation replicas (Ri, i = replica number). Solid and dashed lines represent free (free) and Sec63-bound (Sec63-B) states, respectively. Black star, black triangular and red triangular symbols represent the corresponding values in the experimental cryo-EM structures of Sec complex, idle-state ribosome-Sec61 complex and open-state ribosome-Sec61 complex, respectively. Time-dependent θTM2 profiles during the five MD simulation replicas (B) Sec63-bound state (C) Free state (without Sec63). (TIF) [file pcbi.1008855.s012.tif]

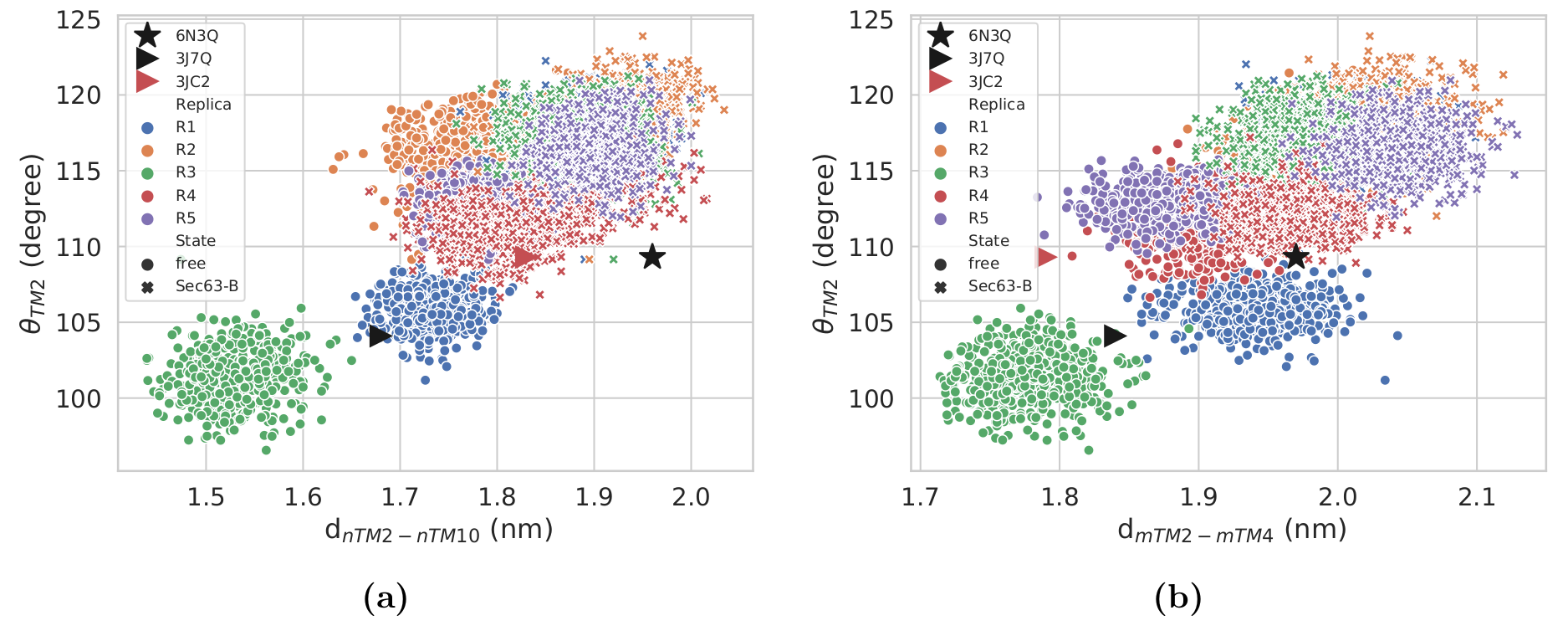

Supplement: S13 Fig — (TIF) [file pcbi.1008855.s013.tif]

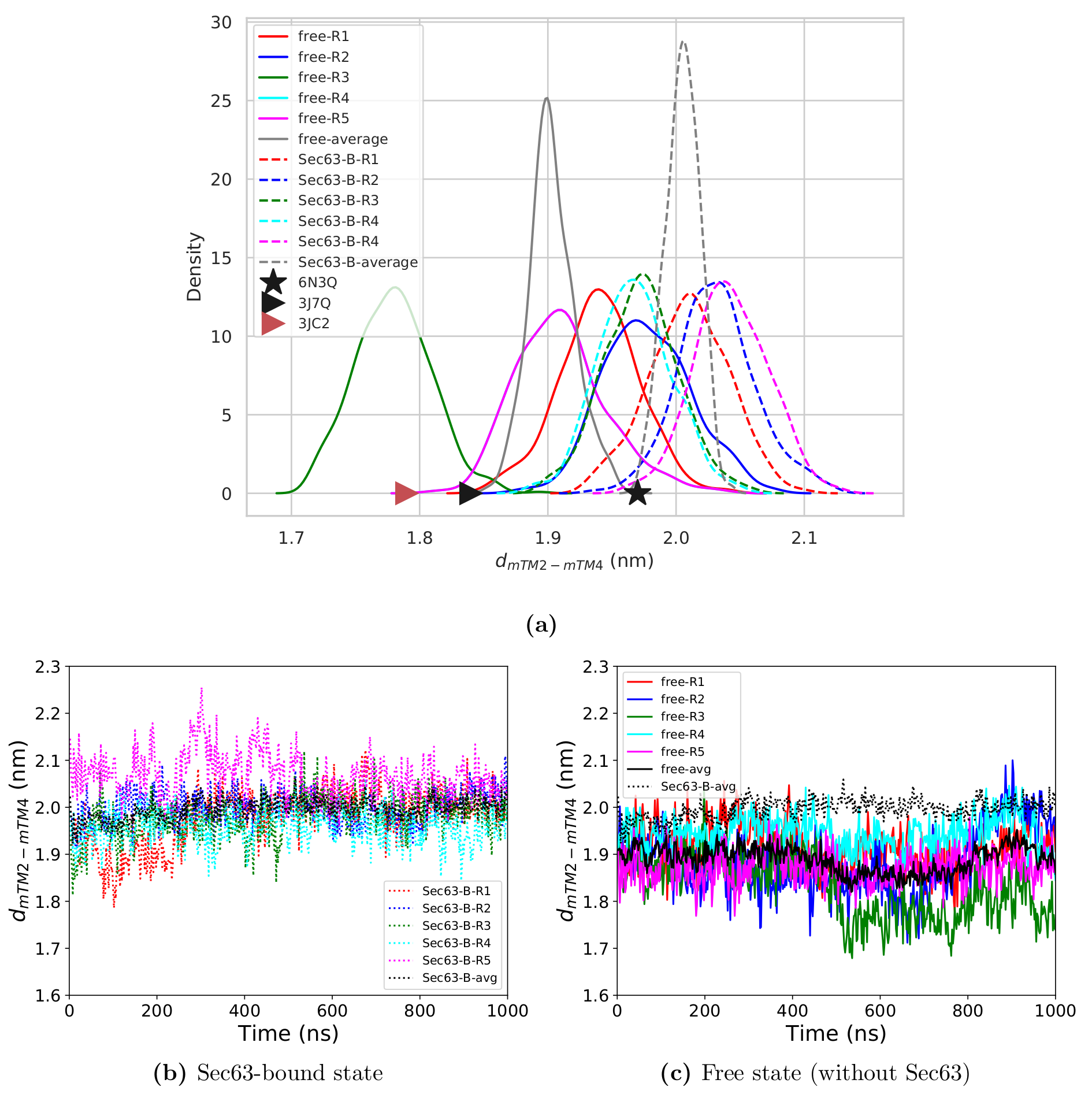

Supplement: S14 Fig — (A) Probability distributions of dmTM2–mTM4 distances obtained from the last 50 ns of five MD simulation replicas (Ri, i = replica number). Solid and dashed lines represent free/without Sec63 (free) and Sec63-bound (Sec63-B) states, respectively. Black star, black triangular and red triangular symbols represent the corresponding values in the experimental cryo-EM structures of Sec complex, idle-state ribosome-Sec61 complex and open-state ribosome-Sec61 complex respectively. Time-dependent dmTM2–mTM4 profiles during the five MD simulation replicas (B) Sec63-bound state (C) Free state (without Sec63). (TIF) [file pcbi.1008855.s014.tif]

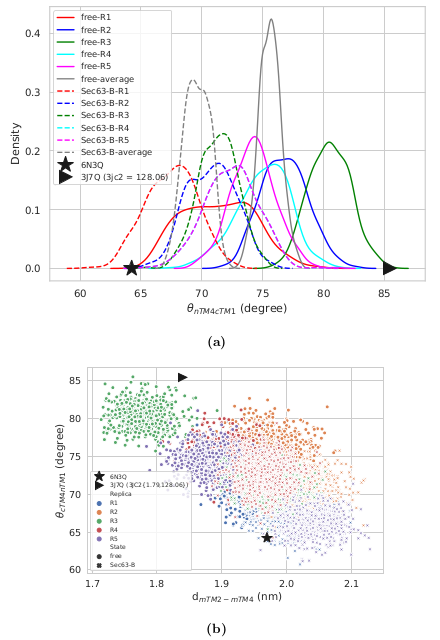

Supplement: S15 Fig — (A) Probability distributions of θcTM4nTM1 angles obtained from the final 50 ns of five independent MD simulation replicas (Ri, i = replica number). Solid and dashed lines represent free (free) and Sec63-bound (Sec63-B) states, respectively. Black star and black triangular marks represent the respective values in the experimental cryo-EM structures of Sec and ribosome-Sec61 (closed state) complexes, (B) Dependence of θcTM4nTM1 on dmTM2–mTM4, during the final 50 ns of the five independent MD simulations (Pearson correlation coefficient -0.70). The values corresponding to the open-state ribosome-Sec61 complex are not included in the plots because their values differ largely. They are only given in the legend. (TIF) [file pcbi.1008855.s015.tif]

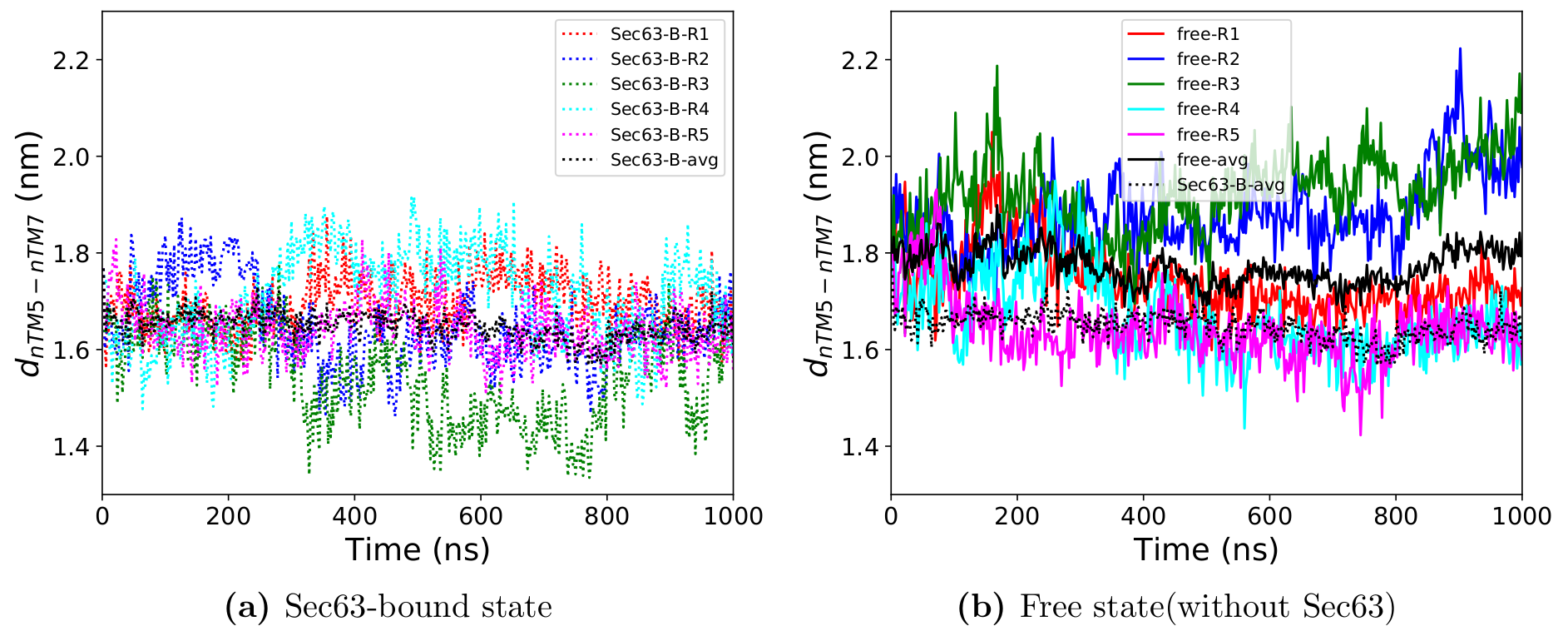

Supplement: S16 Fig — (A) Sec63-bound state (B) Free state (without Sec63). (TIF) [file pcbi.1008855.s016.tif]

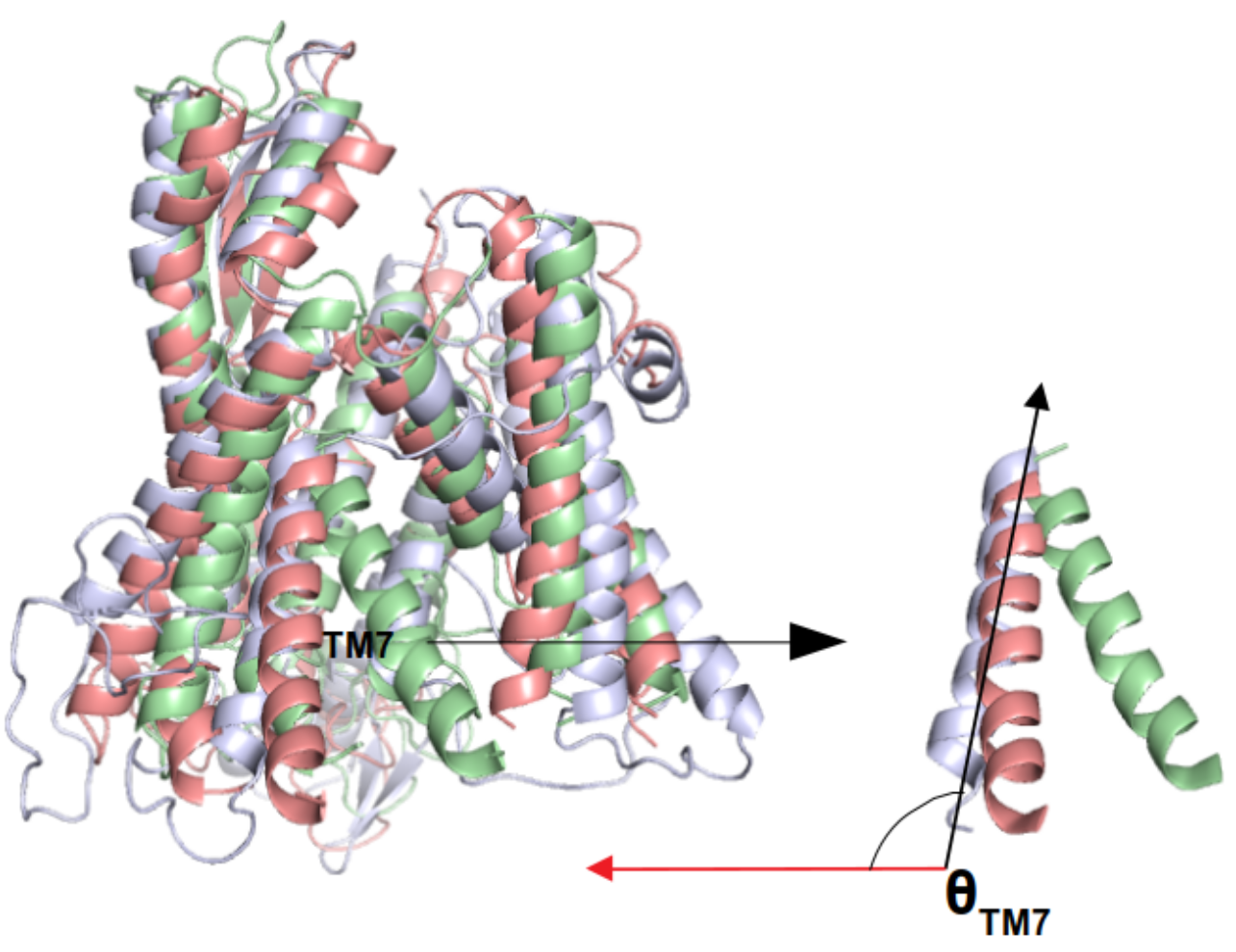

Supplement: S17 Fig — The θTM7 angle describes the orientation of the TM7 helix with respect to the plane of the membrane. The angle, θTM7, is defined as the angle between the TM7 helical axis (black) and the vector parallel to the lipid layer oriented towards the x-axis (red). (TIF) [file pcbi.1008855.s017.tif]

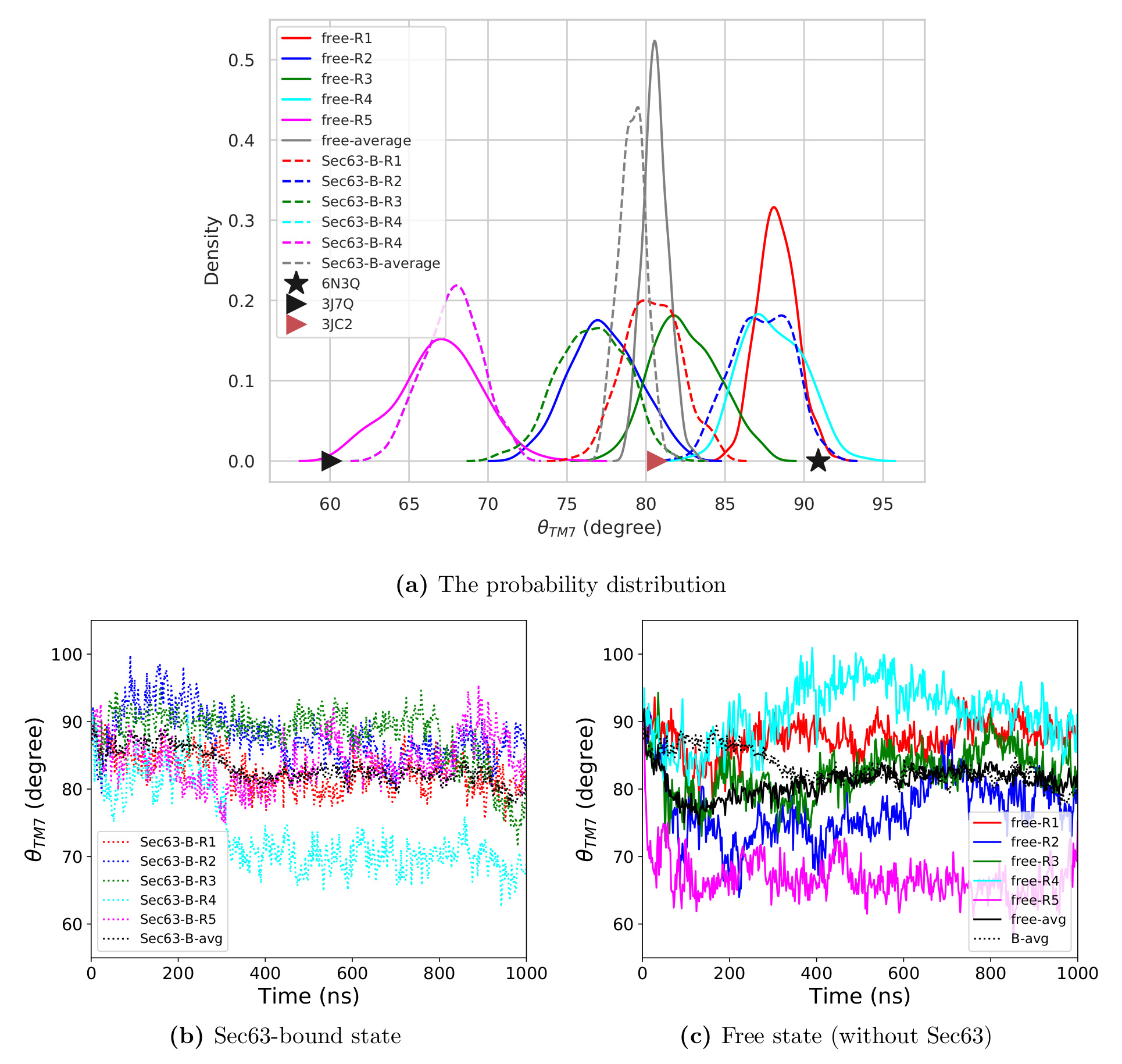

Supplement: S18 Fig — (A) Probability distributions of θTM7 angles obtained from the final 50 ns of five independent MD simulation replicas (Ri, i = replica number). Solid and dashed lines represent free/without Sec63 (free) and Sec63-bound (Sec63-B) states, respectively. Black star, black triangular and red triangular symbolss represent the respective values in the experimental cryo-EM structures of Sec complex, idle-state ribosome-Sec61 complex and open-state ribosome-Sec61 complex, respectively. Time-dependent θTM7 profiles during the five MD simulation replicas (B) Sec63-bound state (C) Free state (without Sec63). (TIF) [file pcbi.1008855.s018.tif]

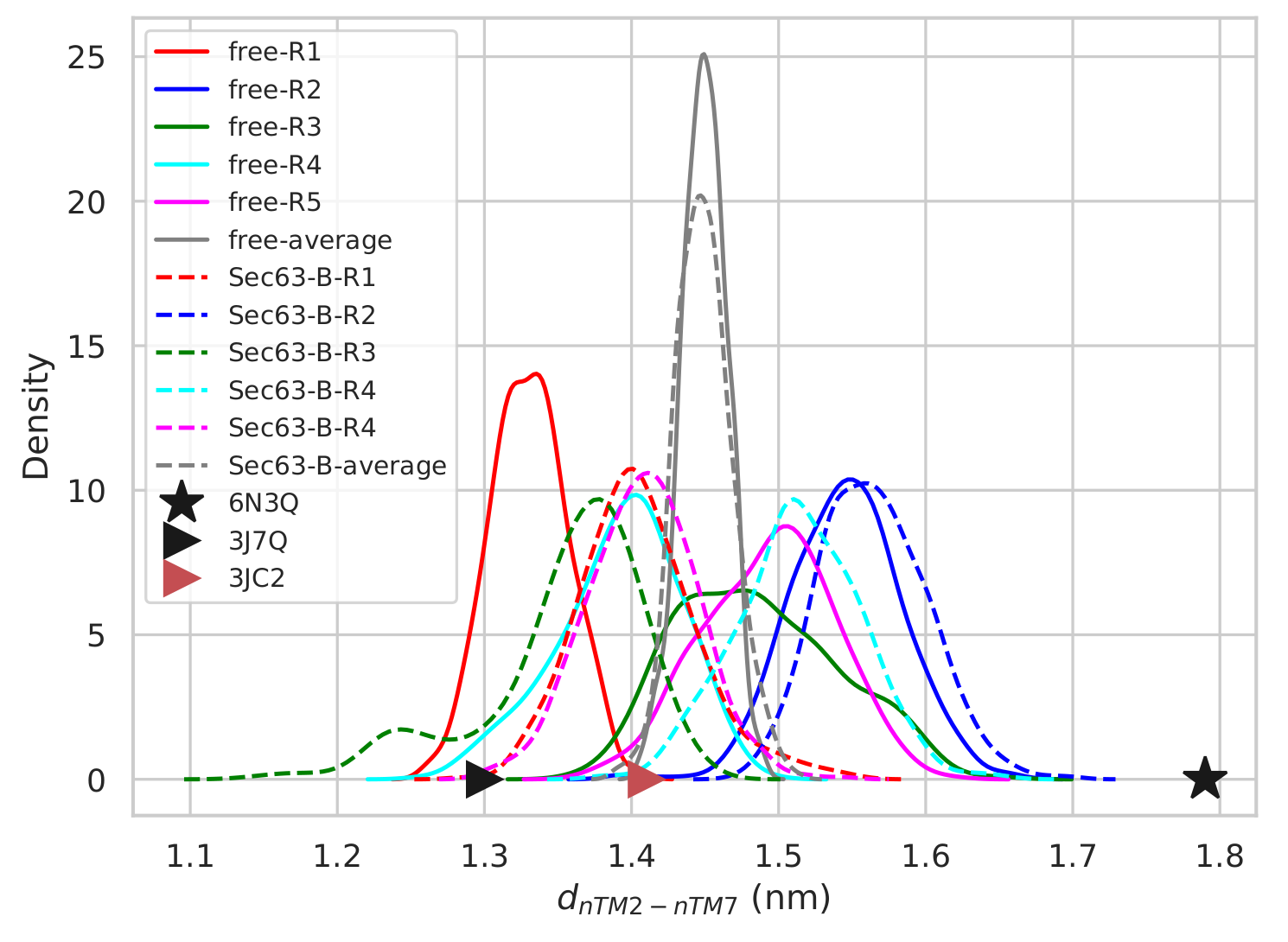

Supplement: S19 Fig — Solid and dashed lines represent simulations of the ‘free’ (free) and ‘Sec63-bound’ (Sec63-B) states, respectively. Black star and triangular symbols represent values in the experimental cryo-EM structures of Sec complex and ribosome-Sec61 complex, respectively. (TIF) [file pcbi.1008855.s019.tif]

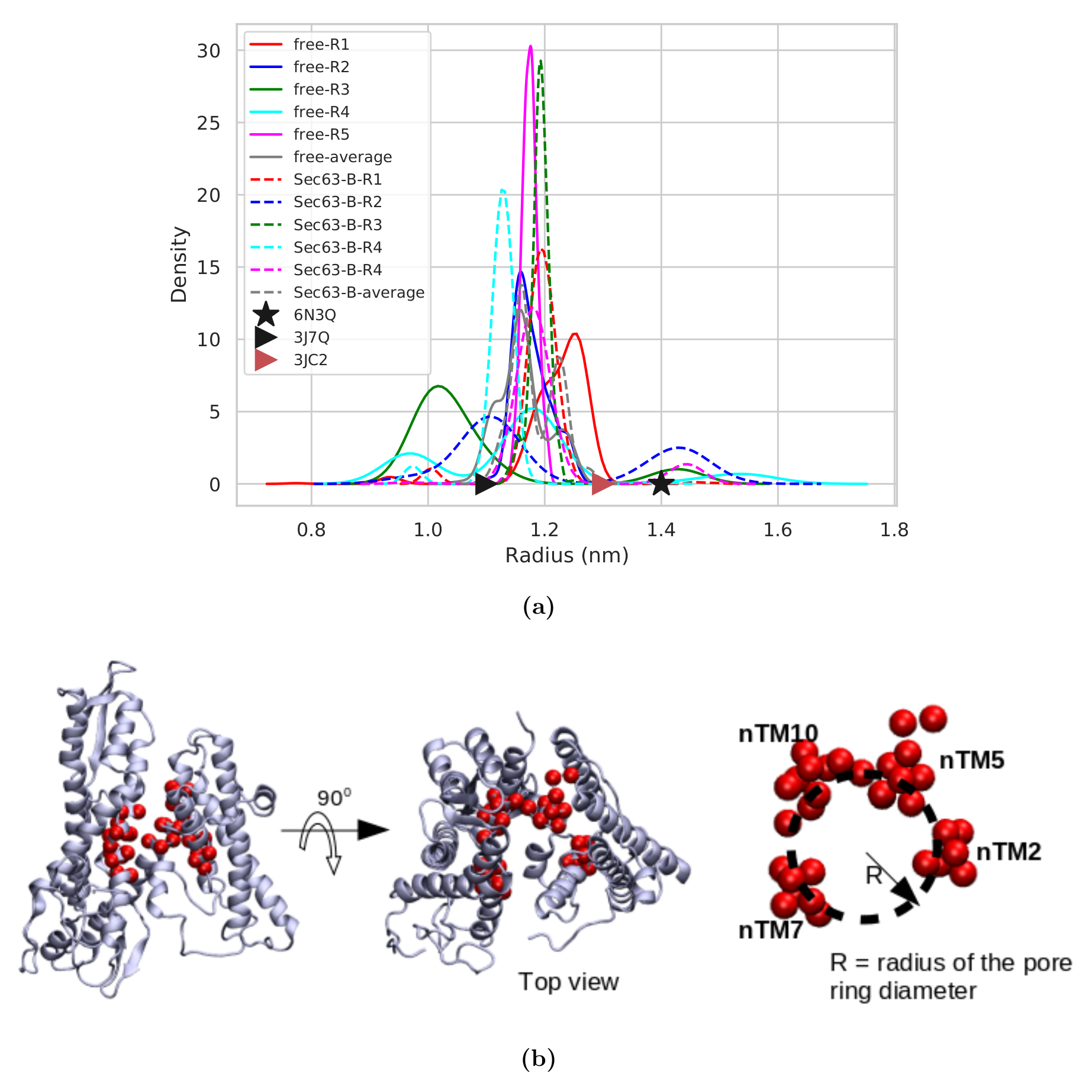

Supplement: S20 Fig — Upper panel: (A) The probability distributions for the radius of the pore-ring region obtained from the last 50 ns of five MD simulation replicas (Ri, i = replica number). Solid and dashed lines represent simulations of the ‘free’ (free) and ‘Sec63-bound’ (Sec63-B) states, respectively. Black star and triangular symbols represent values in the experimental cryo-EM structures of Sec complex and ribosome-Sec61 complex, respectively. Lower panel: (B) The radius of the pore-ring region was calculated using a curve fitting approach. The Cα atoms of the N-termini of TM2, TM5, TM7, TM10 and their contact residues (<0.5 nm distance)(red color sphere) were projected on the lipid bilayer plane (X-Y plane). Then, the equation of a circle was fitted to these projected points. (TIF) [file pcbi.1008855.s020.tif]

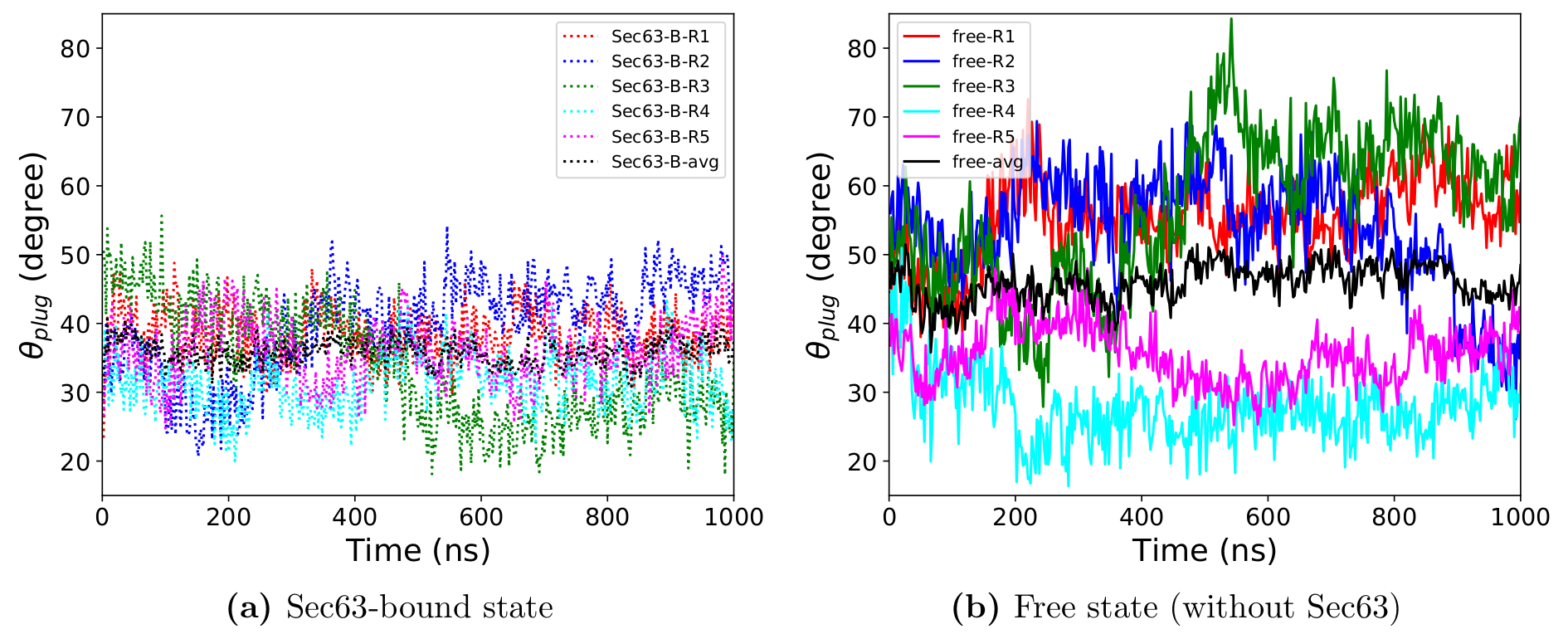

Supplement: S21 Fig — (A) Sec63-bound state (B) Free state (without Sec63). (TIF) [file pcbi.1008855.s021.tif]

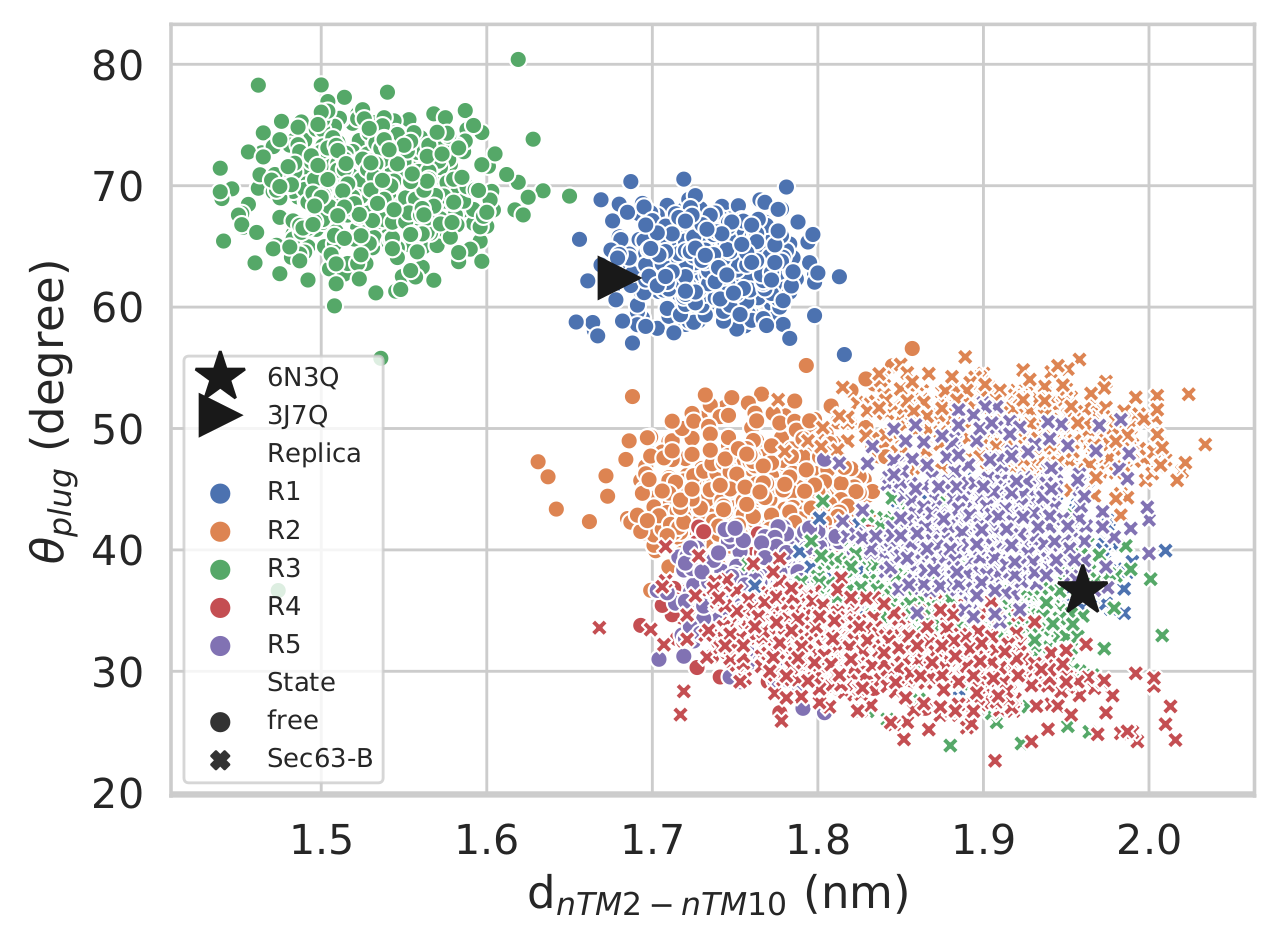

Supplement: S22 Fig — The plug region is missing in the cryo-EM structure of the open-state ribosome-Sec61 complex (PDB ID: 3JC2). (TIF) [file pcbi.1008855.s022.tif]

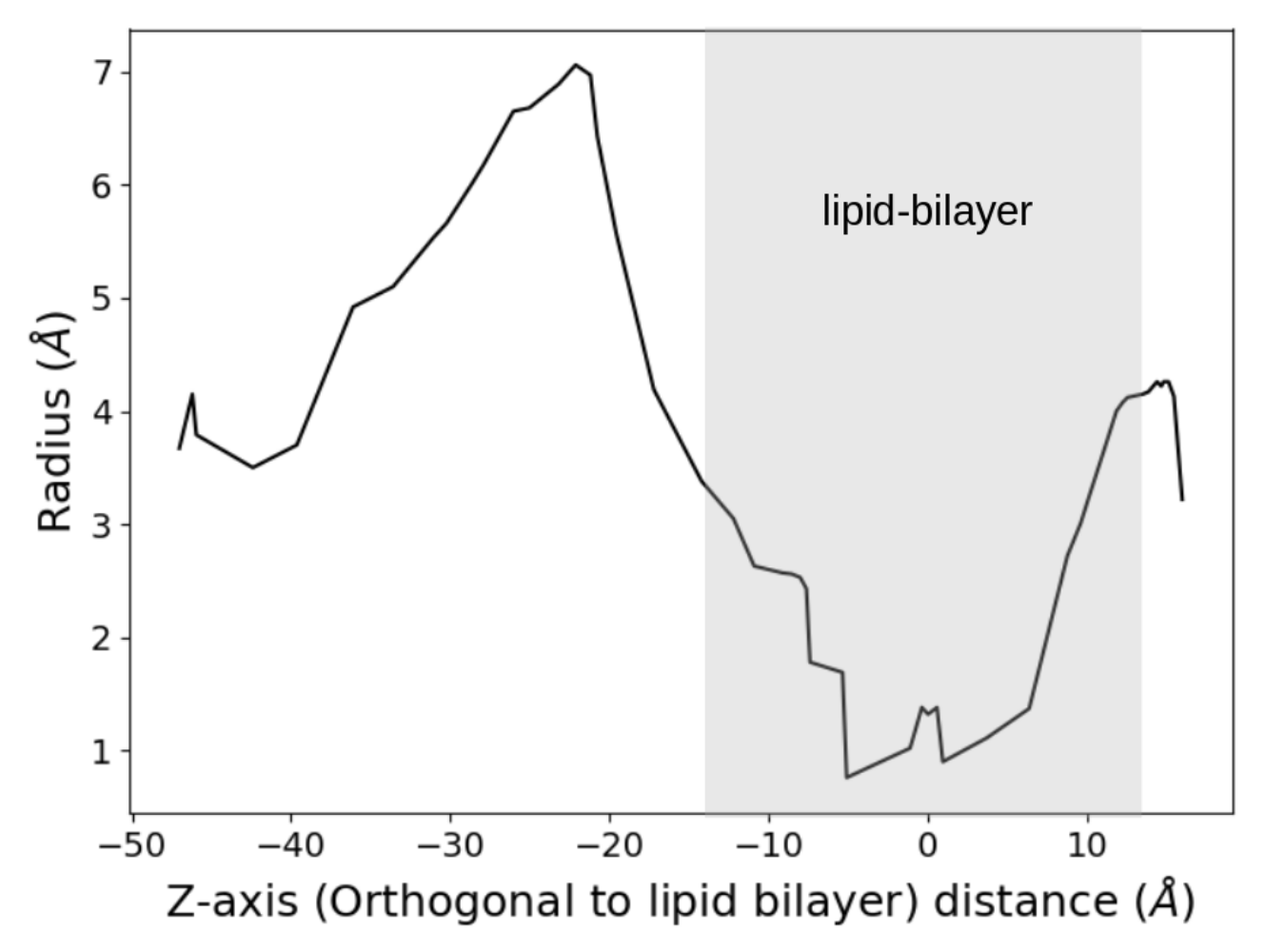

Supplement: S23 Fig — (TIF) [file pcbi.1008855.s023.tif]

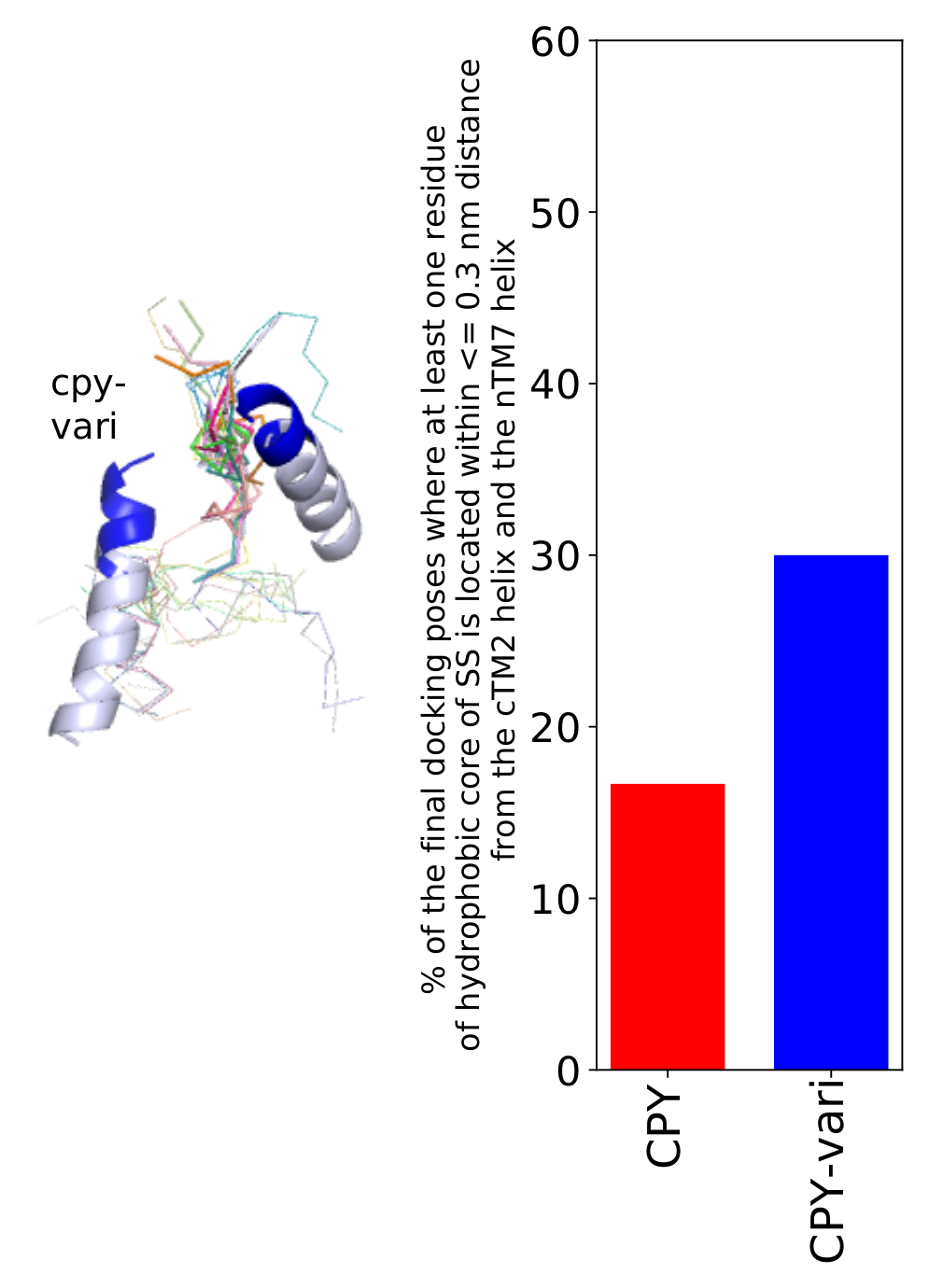

Supplement: S24 Fig — Left-panel: All final docking poses of the CPY-variant (LLTLLLCLLLL) [15] are represented by line model. The final docking poses that satisfy the distance criterion are shown with thicker lines. Sec61, the TM2 helix and the TM7 helix are shown in surface model and cartoon model, respectively. The blue parts of TM2 and TM7 helices represent the C-terminus of TM2 and the N-terminus of TM7. Right-panel: The percentage of the total final docking poses where at least one residue of the hydrophobic core is located near (≤ 0.3 nm residue-residue distance) the C-terminus of TM2 and the N-terminus of TM7. (TIF) [file pcbi.1008855.s024.tif]
