# Supplementary material for: How does Sec63 affect the conformation of Sec61 in yeast?
Source: PLoS Comput Biol. 2021 Mar 29;17(3):e1008855. doi: 10.1371/journal.pcbi.1008855 (PMC8031780; doi:10.1371/journal.pcbi.1008855)
Supplement: Attachment — Submitted filename: Reply_to_reviewer_comments.doc [file pcbi.1008855.s026.doc]

**Reviewer #1:**

This work investigates the effects of bounded Sec63 on the
conformational dynamics of Sec61 channel through MD simulations. The
translocation mechanism of Sec61/SecY complex is very complicated that
involves the cooperation between partners in complex, and remains unclear
so far. In this paper, the authors analysed the position and orientation
changes of involved TM helices by the data comparison. The results will
provide insights on understanding the translocation mechanism. The data of
the simulations is reliable. However, the discussion about the mechanism
how the interaction between Sec63 and TM4 effect the conformational change
of pore ring, lateral gate is not convincing. Few issues need to be
addressed before the manuscript can be accepted.

Major issues:

**1.** For the free simulations (Sec61 complex without Sec63), the authors
simply removed Sec63 from the bound structure (PDB ID: 6N3Q). This will
result in position and orientation changes of pore ring, lateral gate and
plug. In the Fig. 1, the equilibrated structure is reached at around 600 ns
of 1 μs simulations. But the data only the last 50 ns was used to be
analysed.

**Our response:** We agree that the backbone RMSD (Fig. 1 is Fig. 2 in the revised manuscript) and the radius of gyration (Fig. 2 is Fig. 4 in the revised manuscript) have reached plateau values after about 600 ns suggesting that the conformational relaxation of the unbound simulation has taken place by that time. We may further assume that this also applies to local structural features such as the distance and angle measurements of the pore-ring, lateral gate and plug. In principle, one could verify this by extending the MD simulations to considerably longer times and then confirm that nothing changed. Alternatively, we showed that five independent 1 μs long simulations gave fairly consistent results.

We felt that selecting the best-suited analysis window is a balance between (a) averaging over a long time vs. (b) possibly allowing for further structural relaxation before computing a time average. Initially, we went for the second option.

For comparison, as suggested by the reviewer, we now also computed the distribution plots of d_nTM2-nTM10_ (Fig 6 is now Fig. 8 in the revised manuscript), d_nTM5-nTM7_ (Fig 7 is now Fig. 9 in the revised manuscript) and θ_plug_ (Fig 8 is now Fig. 10 in the revised manuscript) using the last 400 ns of the trajectories and added the results to Supplementary Figures S19, S20 and S21 in the revised manuscript.

Supplementary Figure S19 shows that the d_nTM2-nTM10_ distribution computed from the last 50 ns is more narrow and has higher peaks compared to that computed from the last 400 ns. However, the peak positions of the average distributions (grey color) are located at similar values in both cases (free and Sec63-bound) (1.75 Å and 1.88 Å for the last 400 ns and 1.73 Å and 1.88 Å for the last 50 ns, respectively).

In the same manner, the distributions of d_nTM5-nTM7_ (Supplementary Figure S20) obtained from the last 50 ns are narrower than for the last 400 ns. The peak positions are 1.75 Å (free) and 1.65 Å (Sec63-bound) for the last 400 ns and 1.80 Å (free) and 1.65 Å (Sec63-bound) for the last 50 ns. By eye, the free simulations seem better separated from the bound simulations for the last 50 ns than for the last 400 ns. E.g. in the left plot, the red dashed line is slightly shifted to smaller values than the red solid line, whereas the two distributions strongly overlap in the right plot. Also, in the right plot, the light blue dashed line is shifted to larger values that the light blue solid line, which is not the case in the left plot. However, the statistical test gave the same (insignificant) result of p = 0.2 for the left and right plots.

In the distributions of θ_plug._ (see Fig. S21), the peaks of the average distributions of both states (grey lines) are shifted by ~5^o^ to lower values in the last 400 ns compared to the last 50 ns. The overall range of the unbound simulations is 30 – 75 ^o^ in the last 50 ns and 20 – 75 ^o^ in the last 400 ns. The range of the bound simulations is 25 – 55 ^o^ in the last 50 ns and 20 – 50 ^o^ in the last 400 ns. We find that these ranges are quite similar to each other. Also, the statistical p-values are very similar to each other (0.2 vs. 0.2).

In summary, we feel after comparing the statistical distributions of local dynamics measures obtained from the last 400 ns and last 50 ns of the simulation trajectories that the last 50 ns of the trajectories equally well describe the local dynamics as the last 400 ns of the trajectories. Therefore, in the remainder of the manuscript we used the last 50 ns of the 1 μs long MD simulations to analyze the local flexibility of Sec61. We have briefly explained the reason for analyzing the last 50 ns of the trajectories in the revised manuscript (page 5 line 176) and we compare the results of both time windows in the new suppl. figures S19 to S21.

**2.** In the Fig. 3, authors said "the contacts at IS2, IS3 and IS4 were well
maintained, likely due to the regular secondary structure..." (see line
272-279), and the contact at IS1 was not stable that is related to the
missing of some residues. The authors should show the region of bilayer in
Fig. 3(a). The bilayer could make the contact more stable if the
interaction site is located in the bilayer. The fluctuation of Fig. 3(b)
likely due to the solvent too.

**Our response:** We thank the reviewer for this interesting suggestion. After moving two supplementary figures to the main text (as suggested by the second reviewer) figure 3 of the original manuscript is now figure 5 in the revised manuscript.

Please note that figure numbers have been renumbered in the revised manuscript. The locations of the lipid bilayer boundaries are now shown as blue dashed lines in Fig. 5(a) (formerly Fig. 3(a)). The pink rectangular area represents the lipid bilayer (Fig. 5(a)). It turns out that IS1 is located 3.0 nm (V274-G276:Sec61) above the bilayer (phosphate group). We agree with the reviewer that solvent may have an important effect on the flexibility of region IS1. We included this point at line 292 (page 8) in the revised manuscript.

**3.** In the Fig. S4, the RMSF in the region near the IS1 and IS3 should be
magnified to show the significant difference between the bounded and free
state.

**Our response:** As suggested, we magnified the IS1 and IS3 regions of the average (average over five independent simulations) RMSF curve (Fig. S4 is now Supplementary Figure S2 in the revised manuscript) in the revised manuscript. Indeed, these regions show noticeably larger RMSF values in the free state than in the Sec63-bound state.

**4.** In the part of "Resilience of the pore-ring and lateral gate", the
interaction between Sec63 and TM4 of Sec61 makes the θTM2 change. Then, it
makes the dnTM2-nTM10 and dnTM2-nTM4 longer, but the dnTM5-nTM7 shorter. It seems disagree with the cryo-EM structure (line 350). The discussion of
this part is not convincing. Does the Sec63 make the dnTM2-nTM7 longer? How
the Sec63 makes pore and lateral gate wider is still not clear. Figures of
the dnTM2-nTM7 and the diameter of the pore (like Fig. 6,7) should be
helpful to current discussion.

**Our response:** As suggested, we have added plots showing the distribution of d_nTM2-nTM7_ (new Supplementary Figure S22) and the distribution of the radius of the pore-ring (new Supplementary Figure S23) to the revised manuscript.

We found in our work that the interaction between Sec63 and TM4 of Sec61 leads to a change of θ_TM2_ and alters the orientation of TM4. In the absence of this interaction with Sec63 (in the ‘free’ state), the N-terminus of TM2 shifts towards the N-terminus of TM10. This leads to a shortening of d_nTM2-nTM10_ and d_mTM2-mTM4_ and to an increase of d_nTM5-nTM7_. The d_nTM2-nTM7_ and d_nTM5-nTM7_ values represent diagonal distances of the pore-ring (Supplementary Figure S7). Our simulation results demonstrate that the pore-ring conformation is associated with the presence or absence of Sec63. Although, the distance between the N-termini of TM2 and TM7 (distribution d_nTM2-nTM7_) shrinks with respect to the starting conformation in both states (new Supplementary Figure S22). This suggests that the parts of Sec63 included in our simulations are not responsible for the wider d_nTM2-nTM7_ in the cryo-EM structure.

The radius of the pore-ring was calculated using a curve fitting approach. The Cα atoms of the N-termini of TM2, TM5, TM7 TM10 and their contact residues (<0.5 nm distance) were projected on the plane of the lipid bilayer (XY-plane). Then, the equation of a circle was fitted through these protected points (new Supplementary Figure S23 (Lower-panel)). New supplementary Figure S23 shows that the radius shrinks in both conditions (Sec63-bound and free).

Our simulation results only suggest that the conformation of the pore-ring region is associated with Sec63. The results illustrate that Sec63 keeps d_nTM2-nTM10_ wider/longer by interacting with TM4 of Sec61. The change in d_nTM2-nTM10_ may lead to the alteration of the pore region conformation. The distance between the N-termini of TM2 and TM7 (new Supplementary Figure S22) and the radius of the pore-ring (new Supplementary Figure S23) adopted the closed gate conformation in both conditions (Sec63-bound and free). These measurements are done with respect to TM7, which is a part of the lateral-gate. Furthermore, our simulation results demonstrate that the orientation of TM7 is similar in both states (Supplementary Figure S15).

However, the simulations cannot answer what causes the TM7 movement in the simulations of the Sec63-bound state. We assumed that the parts of Sec63 included in our simulations have a negligible influence on the movement of TM7. Recently published cryo-EM structures (Itskanov2021), (Weng2021) shed light on the orientation of TM7 in the Sec complex. They revealed that interactions between the TMs of Sec62 with TM7-Sec61 keep the lateral-gate open in the Sec complex. Besides, Itskanov et al. (Itskanov2021) revealed that the Sec61 channel remains inactive without Sec62. This cryo-EM structure of Sec62 was not available when we conducted our simulations. Hence, the TMs of Sec62 are not present in our Sec63-bound simulations. Now, based on our MD simulations and the new cryo-EM structures, it has become clear that the conformation of the lateral gate and the pore-region are associated with both Sec62 and Sec63.

In the revised manuscript, we clearly explain the conformational changes of the pore-ring region in section “"Resilience of the pore-ring and lateral gate"” (page 12, line 456) and “Discussion” (page 15, line 608).

Minor issues:

Two minor spelling mistakes: line 113: "Ssh1" should be "Sss1". line 655:
"θTM7" should be "θplug".

**Our response:** Thank you for identifying the mistakes. We corrected the typos in the revised manuscript.

**Reviewer #2:**

In this paper, the authors have combined molecular modeling,
i.e., docking and MD simulations, with co-precipitation experiments on the Sec complex especially focusing on Sec61 and Sec63 in yeast. The conformations and
their changes of Sec61 and the effect of the binding of Sec63 to Sec61 were
at the heart of this study. The simulations do show interesting changes in
the plug conformations depending on the presence/absence of Sec63
especially also of the lateral gate. Thus, the present study adds some nice
pieces to the understanding of the translocon and its accessory proteins.

- Add a brief summary of the experimental findings to the Author Summary
and the Conclusion section. A more detailed combination of theory and
experiment in the main text would be helpful for the reader.

**Our response:** We thank the reviewer for this helpful suggestion. We added one sentence in the Conclusion (page 16 line 663) about the results from the co-precipitation experiments for the Sec complex, especially focusing on Sec61 and Sec63 in yeast. Furthermore, we included the experimental finding in the discussion section (page 14 line 544) of the revised manuscript. In the Author Summary, we described the overall contribution of the study instead of individual findings. Thus, we left the Author Summary unchanged.

- I would strongly suggest to include Fig S1 but also Fig S3 to be included
in the main text. The reader needs to get a chance to get acquainted with
the proteins and one cannot expect the reader to look into the SI for
such basic figures. Concerning Fig S1 I would suggest to make the
membrane a bit more visible.

**Our response:** As suggested, we moved Figs. S1 and S3 to the main text. In the revised manuscript, they are now numbered Fig. 1 and Fig. 3. Also, we changed the layout of the membrane in Fig. S1 (Fig. 1 in the revised manuscript) to make the membrane more clearly visible.

- Could the channel in Sec61 be characterized by a radius profile? Is
anything known about its ion conductance?

**Our response:** We thank the reviewer for this suggestion. We added a radius profile of the Sec61 channel (6ND1) that was calculated using the webserver MOLEonline (Prava2018). The radius profiling shows that the radius of the narrowest point of the pore channel is <1.5 Å in the resting condition or closed-state (new Supplementary Figure S24). Further, the channel is blocked by a small α-helical plug from the luminal side. In this static resting conformation, ion movement along the channel pore appears not feasible.

However, (Erdmann2010) demonstrated that the mammalian *C. familiaris* and the *S. cerevisiae* Sec61 channels exhibit almost identical marginal anion selectivity characteristics. In higher eukaryotes (mammalian cells), the heterotrimeric Sec61 complex in the ER membrane provides an aqueous pathway for calcium leakage from the ER to the cytosol (Lang 2011). In yeast, the Sec61 channel may serve as a conduit for small, uncharged peptides like glutathione and the bivalent manganese cation (Witham2020, Ponsero2017).

Other than this, the manuscript is timely and interesting and certainly
deserves publication.

**Additional references:**

Itskanov2021: Itskanov, Samuel, Katie M. Kuo, James C. Gumbart, and Eunyong Park. "Stepwise gating of the Sec61 protein-conducting channel by Sec63 and Sec62." *Nature Structural & Molecular Biology* 28, 162-172 (2021).

Weng2021: Weng, Tsai‐Hsuan, Wieland Steinchen, Birgitta Beatrix, Otto Berninghausen, Thomas Becker, Gert Bange, Jingdong Cheng, and Roland Beckmann. (2020) "Architecture of the active post‐translational Sec translocon." *The EMBO Journal* 40, e105643 (2020).

Lang2011: Lang, S., Erdmann, F., Jung, M., Wagner, R., Cavalie, A., & Zimmermann, R. „Sec61 complexes form ubiquitous ER Ca2+ leak channels“. *Channels*, *5*, 228-235 (2011).

Erdmann2010: Erdmann, F., Jung, M., Maurer, P., Harsman, A., Zimmermann, R., & Wagner, R. “The mammalian and yeast translocon complexes comprise a characteristic Sec61 channel.” *Biochemical and biophysical research communications*, *396*, 714-720 (2010).

Ponsero 2017: Ponsero AJ, Igbaria A, Darch MA, Miled S, Outten CE, Winther JR, et al. “Endoplasmic reticulum transport of glutathione by Sec61 is regulated by Ero1 and Bip”. *Molecular cell*. 67: 962—973 (2017).

Prava2018: Pravda, Lukáš, David Sehnal, Dominik Toušek, Veronika Navrátilová, Václav Bazgier, Karel Berka, Radka Svobodová Vařeková, Jaroslav Koča, and Michal Otyepka. "MOLEonline: a web-based tool for analyzing channels, tunnels and pores (2018 update)." *Nucleic acids research* 46, W368-W373 (2018).

Witham2020: Witham CM, Dassanayake HG, Paxman AL, Stevens KL, Baklous L, White PF, et al., “The conserved C-terminus of Sss1p is required to maintain the endoplasmic reticulum permeability barrier”. *Journal of Biological Chemistry*. 295: 2125—2134 (2020).
